# Mechanisms of Atherosclerosis Induced by Postprandial Lipemia

**DOI:** 10.3389/fcvm.2021.636947

**Published:** 2021-04-29

**Authors:** Yixi Zhao, Longtao Liu, Shengjie Yang, Guijian Liu, Limin Pan, Chun Gu, Yang Wang, Dan Li, Ran Zhao, Min Wu

**Affiliations:** ^1^Comprehensive Department, Guang'anmen Hospital, China Academy of Chinese Medical Sciences, Beijing, China; ^2^Cardiovascular Department, Xiyuan Hospital, China Academy of Chinese Medical Sciences, Beijing, China; ^3^Clinical Laboratory, Guang'anmen Hospital, China Academy of Chinese Medical Sciences, Beijing, China; ^4^Graduate School, Beijing University of Chinese Medicine, Beijing, China

**Keywords:** postprandial lipemia, triglyceride, atherosclerosis, endothelial dysfunction, oxidative stress, inflammation

## Abstract

Postprandial lipemia plays an important role in the formation, occurrence, and development of atherosclerosis, and it is closely related to coronary heart disease and other diseases involving endothelial dysfunction, oxidative stress, inflammation, and other mechanisms. Therefore, it has become a focus area for further research. The studies on postprandial lipemia mainly include TG, TRL, VLDL, CM, and remnant cholesterol. Diurnal triglyceride patterns and postprandial hyperlipidemia are very relevant and are now insufficiently covered. The possible mechanisms between postprandial lipemia and cardiovascular disease have been reviewed in this article by referring to relevant literature in recent years. The research progress on the effects of postprandial lipemia on endothelial function, oxidative stress, and inflammation is highlighted. The intervention of postprandial lipemia is discussed. Non-medicinal intervention such as diet and exercise improves postprandial lipemia. As medicinal intervention, statin, fibrate, ezetimibe, omega-3 fatty acids, and niacin have been found to improve postprandial lipid levels. Novel medications such as pemafibrate, PCSK9, and apoCIII inhibitors have been the focus of research in recent years. Gut microbiota is closely related to lipid metabolism, and some studies have indicated that intestinal microorganisms may affect lipid metabolism as environmental factors. Whether intervention of gut microbiota can reduce postprandial lipemia, and therefore against AS, may be worthy of further study.

## Introduction

Hyperlipidemia clinical diagnosis requires fasting lipemia, but individuals are in the postprandial state for most of the 24 h. Therefore, fasting lipemia cannot comprehensively and accurately reflect lipid metabolism (1, 2). The viewpoint that atherosclerosis (AS) is a postprandial phenomenon was proposed by Zilversmit as early as 1979 (3). With the growing awareness of postprandial lipemia, its dynamic change is thought to be closely related to AS. Long-term disorders of lipid metabolism, endothelial dysfunction, and hypercoagulability can occur through postprandial hyperlipidemia, and it participates in AS-related processes (4). As an independent predictor of cardiovascular disease (CVD), postprandial triglyceride (TG), which has vital clinical implications, is a worthwhile contender as a potential therapeutic target (5, 6). The regulation of exogenous chylomicrons (CMs) is closely related to the production and secretion of endogenous very low-density lipoprotein-TG (VLDL-TG) (4). Therefore, improvement of postprandial status by intervening postprandial lipid metabolism could be a measure to reduce the risk of CVD. Our objectives were to (1) review existing literature and study the causes of postprandial hyperlipidemia, (2) review the influence of postprandial lipid metabolism on the formation of AS and study the relationship between postprandial lipemia and AS, and (3) review the effects of postprandial lipemia on endothelial function, oxidative stress, inflammation, apoptosis, endoplasmic reticulum stress, and mitochondrial dynamics, as well as analyze their mechanisms and explore potential interventions.

## Postprandial Lipemia and Postprandial Hyperlipidemia

Dyslipidemia generally refers to all kinds of serum dyslipidemia, including total cholesterol (TC), TG, and low-density lipoprotein-cholesterol (LDL-C), and as initial factors leading to AS (7). Postprandial lipemia refers to the fluctuation of blood lipid levels between the period after eating and the premeal level and is characterized by rising levels of TG-rich lipoproteins (TRLs) (8). TG mainly comes from the decomposition of fat in the diet and can be generated by metabolic activities in the body. VLDL and CM are the main forms of plasma TG, which are collectively termed TRLs (9). The roles of TG and TRL in the pathogenesis of CVD were reviewed by the European Atherosclerosis Society Consensus Panel, which considered that the number of TRLs plays an important role in the relationship between TG and CVD, and apoB100-containing TRLs are a pathological factor promoting the formation of hyperlipidemia (10). High TG level is an important residual risk factor for coronary heart disease (CHD), and postprandial hyperlipidemia refers to the increase of TG-rich CM remnants and persistent hypertriglyceridemia (11). The study of postprandial lipemia parameters in patients with diabetes and prediabetes found that postprandial TG and TG/HDL-C were better than the corresponding fasting parameters in reflecting dyslipidemia and suggesting cardiovascular status (12). Progressive postprandial hyperlipemia is related to obesity, atherosclerosis, and other diseases (13, 14).

The modern diet consists of three to five feeding times a day, and individuals spend a significant amount of time in a non-fasting and postprandial state, meaning the levels of CM, VLDL, and other TRLs and their remnants are enhanced, which leads a continually fluctuating level of blood lipid (15). The postprandial metabolic status can be better reflected by postprandial TG concentration, and with the combination of fasting TG concentration, the degree of exposure of total TG in the arterial wall within 24 h can be described more comprehensively and accurately (16). TG reflects the burden of remnant cholesterol (RC) and can be used as its marker. A Mendelian randomized design examining the relationship between non-fasting RC and ischemic heart disease found that when non-fasting RC increased by 1 mmol/L (39 mg/dl), the causal risk of ischemic heart disease increased by 2.8 times and was not associated with decreased HDL cholesterol (17). This indicates that the increase of cholesterol content in TRL particles accelerates the development of ischemic heart disease. Teno et al. (18) observed serum lipid levels and carotid artery media thickness in 61 patients with type 2 diabetes (T2D) and found that the minimum value of postprandial TG-induced AS was 2.27 mmol/L, suggesting that this concentration level could be diagnosed as postprandial hyperlipidemia. A few aspects of non-fasting and postprandial TG were discussed, and some recommendations were proposed by a panel of clinicians and scientists. It was pointed out that TG levels <2.5 mmol/L at any time after a meal can be regarded as the ideal level of postprandial TG (19).

Circadian rhythm is essential for maintaining life cycle. The diurnal pattern of TG and gut microbiota changes after a high-fat diet, which affects physiological homeostasis (20). Glucocorticoid receptors (GC) regulate circulating TG differently during non-fasting and fasting. Animal experiments showed that the daytime TG level of mice decreased after hepatocyte-specific GC was knocked out, suggesting that circadian rhythm and daytime TG mode affect metabolism (21). The relationship between diurnal TG pattern and postprandial hyperlipidemia has not been reported enough, which is worthy of further study.

## Postprandial Lipemia and Atherosclerosis

Plasma contains hepatogenic atherogenic lipoprotein in the fasting state and enterogenic atherogenic lipoprotein in the non-fasting state. Individuals spend more than 18 h a day in a non-fasting state (22). Thus, lipid metabolism cannot be accurately reflected by fasting lipemia. Postprandial lipemia independently predicts the risk of CVD and likely better reflects the plasma atherogenic lipoprotein levels (9, 23). Non-fasting TG has been shown to be superior to fasting TG in predicting cardiovascular risk (24). A study comparing the composition and size of lipoprotein showed that postprandial VLDL particles measured directly in healthy individuals were significantly increased, which may promote arteriosclerosis of postprandial hyperlipidemia (25). Recognition of non-fasting lipid profiles has been expressed by relevant associations, guidelines, and statements in the United States, Canada, the United Kingdom, and other countries (26–29).

A positive association between postprandial TG levels and CHD was proposed in 1992 (30). Case–control studies have also shown that elevated TG levels in postprandial lipemia are associated with AS and CVD (31). Studies based on postmeal status have shown that abnormal lipoproteins increase the risk of AS, of which TG metabolic capacity is a key factor (32).

In a study of 30 patients with stable CHD who underwent catheter intervention, thin-cap fibroatheroma (TCFA) detected by multivessel examination using optical coherence tomography was found independently associated with postprandial apoB48 (33). ApoB48 is the major apolipoprotein in chylomicron, and postprandial delayed hyperchylomicronemia is considered to be related to the destruction of the TCFA composed of the large lipid core under the fibrous cap in the pathogenesis of acute coronary syndrome (ACS), consequently (34).

Lipoproteins with high atherogenic effects, such as TRLs, are closely related to CHD. Therefore, it is considered that the determination of postprandial lipemia is more sensitive and prominent than fasting lipids in the risk assessment of CHD (35, 36). The clinical research protocols for postprandial lipemia have not been unified, and the evaluation of related animal models is still being explored, so the management guidelines for postprandial lipemia still need to be improved (37).

## The Related Mechanism of Postprandial Lipemia-Induced Atherosclerosis

### Postprandial Dyslipidemia, Endothelial Dysfunction, and Atherosclerosis

Endothelial dysfunction is considered to be an early and reversible predictor of AS and is induced by elevated TG in circulating lipids of postprandial hyperlipidemia (38). The endothelium plays a role in preventing the formation and development of AS through platelet aggregation, vascular tension, coagulation, and fibrinolysis (39). The activation of white blood cells and oxidative stress produced by monodietary fat meal may lead to endothelial dysfunction and form the early stages of AS (40, 41).

A large number of studies have shown that flow-mediated vasodilation (FMD) is affected by postprandial lipemia. It was found that TG increased but FMD decreased postprandially. The increment area under the curve (iAUC) of postprandial TG was negatively correlated with the decrement area under the curve of postprandial FMD, indicating that FMD had transient and significant injury in the postprandial phase and was closely related to postprandial TG (42–44). The imbalance between the vasodilator and vasoconstrictor is one of the characteristics of endothelial dysfunction, and the decrease of the vasodilator is mainly caused by the postprandial decrease of NO and the increase of oxidative stress (45). Robinson Ramirez-Velez recruited 14 healthy men, who were between 18 and 25 years of age, to participate in the high-fat meal (HFM) test. The TC and TG levels were detected, and endothelial function was assessed by measuring serum nitrite/nitrate levels (NO_2_/NO_3_). It was found that 2 h after the meal, the TG levels increased (*p* = 0.04). Endothelial function decreased to 3.3 ± 0.5% (*p* = 0.03) compared with 5.9 ± 1.1% at baseline, and FMD reduced from baseline level of 5.9 ± 1.1 to 3.3 ± 0.4% (*p* = 0.04), manifesting that postprandial lipemia leads to endothelial dysfunction by changing circulating blood lipids (46).

**Figure 1:**
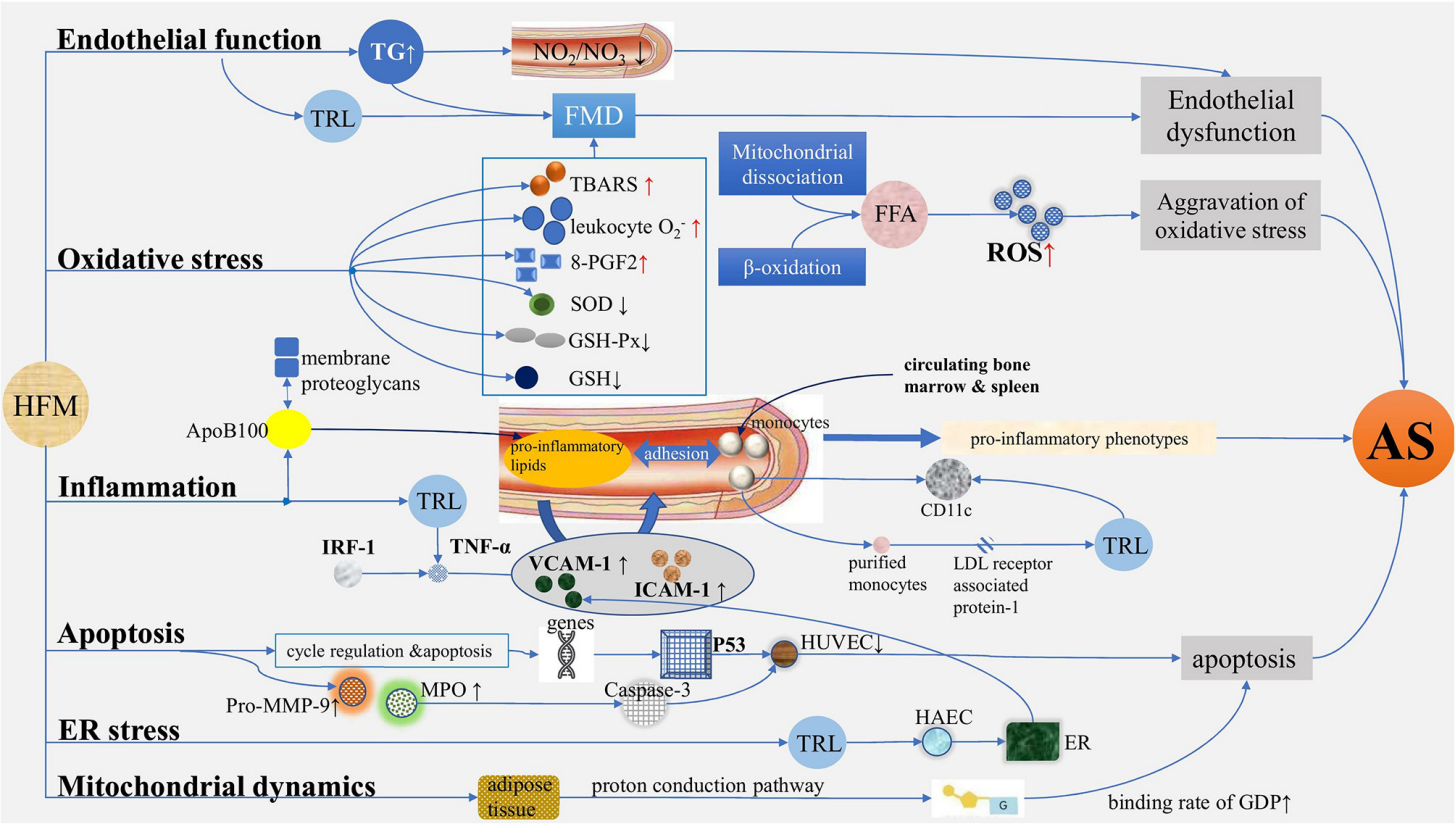
Mechanisms of atherosclerosis induced by postprandial lipemia. (1) Endothelial function: TG increased after HFM, while serum nitrite/nitrate levels and FMD decreased, suggesting endothelium dysfunction. (2) Oxidative stress: In terms of oxidative stress, TBARS, leukocyte ${\text{O}}_{2}^{-}$, and 8-PGF2 increased, and SOD, GSH-PX, and GSH decreased after HFM. ROS are produced in large quantities due to the increased levels of free fatty acids mediated by mitochondrial dissociation and β-oxidation. (3) Inflammation: ApoB100-containing lipoproteins enter into the arterial subendothelium, thus generating proinflammatory lipids, promoting the expression of adhesion factors such as, VCAM-1 and ICAM-1, which induce the augmentation of adhesion interaction with monocytes in turn, generating proinflammatory phenotypes and AS; TRL isolated from human plasma after HFM regulated TNF-α-induced VCAM-1 expression by regulating IRF-1-dependent transcription mechanism. The expression of CD11c on monocytes increased after a meal, and the purified monocytes internalized TRL isolated from postprandial blood through LDL receptor associated protein-1, which also caused the upregulation of CD11c. (4) Apoptosis: With the relation of cell cycle regulation and apoptosis, a group of genes of HUVEC differentially expressed in serum of postprandial hyperlipidemia after a meal, causing proliferation of HUVEC, significantly decreased and may be achieved by activating p53 network; the activities of pro-MMP-9, MPO, and caspase-3 increased significantly after a meal, showing apoptosis effect. (5) ER stress: The expansion of ER was significantly increased by HAEC treated with postprandial TRL, and the production of TRL increased the adhesion of VCAM-1-dependent monocytes to inflammatory endothelium, indicating ER stress plays a role in the regulation of VCAM-1 transcription in endothelial cells during the process of AS. (6) Mitochondrial dynamics: The binding rate of GDP increased by 85% after HFM, and the increase was the largest in early postprandial period. TG, triglyceride; HFM, high-fat meal; FMD, flow-mediated vasodilation; TBARS, thiobarbiturate reactants; 8-PGF2, 8-external prostaglandin F2; SOD, superoxide dismutase; GSH-PX, glutathione peroxidase; ROS, reactive oxygen species; VCAM-1, vascular cell adhesion molecule-1; ICAM-1, intercellular adhesion molecule-1; AS, atherosclerosis; TRL, TG-rich lipoprotein; LDL, low-density lipoprotein; HUVEC, human umbilical vein endothelial cell; ER, endoplasmic reticulum; HAEC, human aortic endothelial cell.

Shafieesabet et al. explored the acute effect of HFM on the changes of peripheral vascular endothelial function in patients with heart failure with reduced ejection fraction (HFrEF). The peripheral vascular endothelial function of patients was evaluated by EndoPAT 2000 technology at baseline and 1, 2, 3, and 4 h after oral TG load using reactive hyperemia index and pulse wave amplitude. Compared with obviously postprandial vascular dilation in healthy individuals, HFrEF patients did not show the same changes. It is suggested that peripheral vascular endothelium function was damaged postprandially in HFrEF patients (47). Postprandial TRL was considered to be the cause of AS. Postprandial TRL is thought to be a contributing factor to AS; Whisner et al. assessed the effects of meal with different dietary components on endothelial function in 10 adolescents aged 10–17 years. TG and FMD were measured at baseline and 4 h postprandially. The results showed that TG at 4 h was negatively correlated with FMD after high-fat and low-fiber meal (β = −0.087; 95% CI = −0.138~-0.037; *p* = 0.001), meaning the importance of the reverse relationship between TRL and FMD in AS (48) (Figure 1).

### Postprandial Dyslipidemia, Oxidative Stress, and Atherosclerosis

Modern lifestyles lead to an exaggerated and prolonged postprandial metabolism, oxidation, and immune imbalance for most of the day, known as postprandial oxidative stress, which promotes the development of CVD (49).

There is a strong relationship between the pro-oxidative load caused by absorption of postprandial lipid hydroperoxides and the impairment of endothelium-dependent vasodilation induced by postprandial lipemia (50). Reactive oxygen species (ROS) are produced in large quantities under physiological and pathological conditions such as inflammation, ionizing radiation, and ischemia, due to the increased levels of free fatty acids mediated by mitochondrial dissociation and β-oxidation after meals (51). Postprandial TRL lipolysis products were found to activate mitochondrial ROS, damage human brain microvascular endothelial cells *in vitro*, and promote AS (52, 53). Control of oxidative stress is important for the stability of endothelial function (54).

Tsai et al. conducted a clinical trial evaluating the effect of postprandial lipemia on oxidative stress in 16 healthy subjects without coronary risk factors. Serum TG, plasma glutathione peroxidase (GSH-PX) levels, and urine excretion of 8-external prostaglandin F2 (8-PGF2) were monitored, and brachial artery FMD was evaluated by high-resolution ultrasound before and 2, 4, and 6 h after the standard HFM. The results showed that after HFM, TG increased significantly; GSH-PX level decreased, declaring increased antioxidant enzyme consumption. The excretion of 8-PGF2 increased, indicating the increase of oxidative modification products. FMD decreased, suggesting that oxidative stress was enhanced after meal and endothelial dysfunction occurred in healthy individuals (55). Bae et al. (56) carried out HFM tests in 11 healthy people. Serum TG, PMA-activated leukocyte ${\text{O}}_{2}^{-}$ production, and FMD were detected at baseline and 2 h after meal. Postprandial serum TG and PMA-activated leukocyte ${\text{O}}_{2}^{-}$ production were significantly higher than before meal, whereas the postprandial FMD level was lower than before meal, which suggested that an acute increase in postprandial TG may cause endothelial dysfunction through elevated oxidative stress. Neri et al. conducted an HFM test in 40 patients with T2D, 40 patients with impaired glucose tolerance (IGT), and 40 healthy subjects. Serum TG, markers of systemic oxidative stress, and endothelial function were measured before and 2, 4, and 8 h after a meal. The results showed that the postprandial TG in each group was higher than the baseline, and the postprandial TG level of T2D patients was significantly higher than that of the IGT group, which was higher than that of the control group. Postprandial TG was significantly enriched in T2D patients and IGT patients at 4 h, while TG was enriched at 2 and 4 h in healthy subjects. The urinary level of free 8-iso-prostaglandin F_2α_ (8-iso-PGF_2α_; F2-isoprostanes) in each group was higher than that in the fasting state. FMD in T2D and IGT patients decreased significantly at 4 h, demonstrating that the changes of postprandial lipemia were closely related to the systematic markers of oxidative stress and led to the impairment of vasodilation function (57). It was found that thiobarbituric acid reactive substances (TBARS) increased and red blood cell reducing glutathione (GSH) and superoxide dismutase (SOD) decreased after HFM in patients with T2D with macrovascular complications, illustrating that damage to antioxidant capacity might lead to endothelial dysfunction (58). Fat load leads to aggravation of oxidative stress and augment of postprandial lipemia, both of which promote the development of CVD (59).

This evidence suggests that oxidative stress induced by postprandial lipemia may have detrimental effects on multiple stages of AS, in addition to direct damage to endothelial function (Figure 1).

### Postprandial Dyslipidemia, Inflammation, and Atherosclerosis

The instability of lipid metabolism and chronic postprandial inflammation are the underlying pathogenesis of AS-induced CVD (41). ApoB100-containing lipoproteins enter the arterial subendothelium and are retained by interaction with membrane proteoglycans through charge-based interactions and sphingolipases. The lipoproteins then become proinflammatory lipids by modification of aggregation, saccharification, and oxidation, promoting the expression of adhesion factors such as, vascular cell adhesion molecule-1 (VCAM-1) and intercellular adhesion molecule-1 (ICAM-1), inducing the augmentation of adhesion interaction of monocytes from circulating bone marrow and spleen, generating proinflammatory phenotypes, and producing the early stages of AS (60).

Inflammation plays a key role in the activation of endogenous and adaptive immune pathways in the pathophysiological process of AS. Vascular inflammation is caused by the accumulation and modification of atherosclerotic lipoprotein in the subendothelium, which leads to the changes in vascular structure, the development of AS, and the pathological formation of CVD (60). Postprandial lipemia may be the main factor leading to inflammation and it affects endothelial function by changing the state of inflammation (61, 62). A single feeding is thought to have the ability to produce a transient and low-intensity inflammatory response (63). The augment of postprandial TG is related to the upregulation of proinflammatory genes in endothelial cells, the increased expression of leukocyte activation markers, and the involvement of the complement system. These processes together constitute part of postprandial inflammation and are considered to be the potential mechanism of promoting AS (64–66).

DeVerse et al. examined IRF-1 and TNF-α-induced VCAM-1 in human aortic endothelial cells (HAECs) stimulated by TRL through immunofluorescence in a microfluidic device. It was found that TRL isolated from human plasma after HFM regulated TNF-α-induced VCAM-1 expression by regulating the IRF-1-dependent transcription mechanism, which indicated that postprandial TRL plays a direct role in regulating the expression of IRF-1 and downstream inflammatory reaction, affecting endothelial function and producing an atherogenic effect (67). The adhesion of monocytes in the arterial wall is associated with AS. Gower et al. detected the blood of healthy subjects with standardized HFM by flow cytometry and found that the expression of CD11c on monocytes increased at 3.5 h after a meal, and the degree of upregulation was related to postprandial TG. The purified monocytes internalized TRL isolated from postprandial blood through LDL receptor associated protein-1, which also caused the upregulation of CD11c. These results suggest that mononuclear cells in the postprandial state can internalize lipids, upregulate CD11c, increase adhesion to VCAM-1, and increase the risk of atherosclerosis (68). Gorzelak-Pabis et al. studied the effects of a single high-fat diet on the barrier function and inflammatory status of human umbilical vein endothelial cells (HUVECs). Serum from healthy volunteers was extracted before and 3 h after a meal to induce HUVECs. The integrity of HUVECs was measured in the RTCA-DP-xCELLigence system. Fasting and postprandial TG were measured. The expression of proinflammatory cytokine mRNA was detected by RT-PCR. Postprandial TG level increased, and postprandial serum decreased the integrity of HUVECs. The mRNA expression of IL-33, IL-32, MCP-1, and CX3C chemokines was significantly increased, which revealed that a single HFM could aggravate the inflammatory process and destroy the endothelial barrier (69). Herieka et al. reviewed 57 studies on HFM-induced postprandial inflammation and found that the increase in systemic inflammation promoting the development of AS can be explained, at least in part, by frequent consumption of excess dietary fat. The results of postprandial inflammation markers were compared with the highly consistent low-grade human endotoxemia model, which showed that plasma-borne inflammatory markers such as, cytokines and soluble adhesion factors did not continuously increase after HFM, while the level of proinflammatory leukocyte surface markers increased in almost all measured studies. It was proposed that the study of inflammation induced by postprandial lipemia could be carried out by focusing on leukocytes in the future (70). Recently, a study has shown that the formation of proatherogenic endothelial phenotype is promoted by postprandial metabolism changes in TRL characterized with oxylipin signature in dyslipidemic subjects (71).

The mechanism of atherosclerotic formation by postprandial lipids and lipoproteins is directly or indirectly involved through partial induction of the inflammatory state. Therefore, anti-inflammatory therapy may further reduce the risk of atherosclerotic CVD in addition to reducing cardiovascular risk factors and improving lipid-lowering therapy (72) Figure 1.

### Other Mechanisms

#### Apoptosis

Dejeans et al. cultured HUVECs in a medium containing 10% serum extracted from healthy subjects before and after HFM to explore the effect of postprandial lipemia on vascular endothelial cells. It was found that the proliferation of HUVECs in postprandial hyperlipidemia was significantly lower than that before meal. The transcriptomic profiles of endothelial cells were changed, among which, a group of genes differentially expressed before and after meal was related to cell cycle regulation and apoptosis, and may be achieved by activating the p53 network. It was indicated that the transcription of genes related to apoptosis in vascular endothelial cells after exposure to postprandial hyperlipidemia may promote vascular dysfunction (73). Spallarossa et al. carried out an HFM test in 15 people between 20 and 45 years of age. Blood samples were collected before and 1, 2, and 4 h after a meal. Plasma TG, myeloperoxidase (MPO), and matrix metalloprotein-9 (MMP-9) activities were measured. HUVECs were cultured in human serum and annexin PI staining and caspase-3 activity were detected by flow cytometry. The TG, activities of MPO, and pro-MMP-9 increased significantly at 4 h after meal. Postprandial serum significantly increased the percentage of annexin-positive HUVECs and the activity of caspase-3. These results indicate that HFM can promote the apoptosis of endothelial cells, and the apoptosis rate is closely related to the increase in MPO and pro-MMP-9 activity, suggesting a possible mechanism of endothelial injury induced by postprandial lipemia (74) (Figure 1).

#### Endoplasmic Reticulum Stress

As previously reported, HAEC can induce an atherosclerotic state by TRL isolated from human blood after HFM. Wang et al. explored the role of endoplasmic reticulum (ER) stress in TRL metabolism and VCAM-1 regulation. Blood samples were collected before and 3.5 h after HFM from healthy subjects and hypertriglyceridemia patients. TRL was separated from postprandial blood samples by ultracentrifugation. HAECs were cultured in Petri dishes for 4 h. The HAEC endoplasmic reticulum morphology was imaged by confocal microscopy and immunofluorescence staining of calreticulin. It was found that the expansion of ER was significantly increased by HAEC treated with postprandial TRL, and the production of TRL after meals increased the adhesion of VCAM-1-dependent monocytes to the inflammatory endothelium. It was indicated that ER stress plays a role in the regulation of VCAM-1 transcription in endothelial cells during the process of atherosclerosis induced by postprandial lipemia (75) (Figure 1).

#### Mitochondrial Dynamics

The mitochondria are thought to be the cell's power source because they produce the metabolic fuel ATP (76). Inflammation, apoptosis, and oxidative stress can promote the development of atherosclerotic plaques, and dysfunction caused by mitochondrial injury can promote these processes (77). Animal experiments showed that the binding rate of mitochondrial guanosine diphosphate (GDP) in brown adipose tissue was measured at one or more time points after the dietary test to indicate the uncoupling respiration rate, and Na^+^-K^+^-ATPase activity represented the coupled rate. It was found that the binding rate of GDP increased by 85% after the test, and the increase was the largest in the early postprandial period. It significantly decreased at 10 h after meal. This indicates that the proton conduction pathway in brown adipose tissue can be activated after a single meal (78) (Figure 1).

## Interventions on Postprandial Lipemia

### Non-medicinal Intervention

#### Diet

Postprandial lipemia is closely related to dietary habits and meal composition (99). The generation of CM and the change in postprandial lipemia were highly affected by the single meal nutrition structure (100). The release of fatty acids from solid food was lower than that from liquid or semisolid food, and the postprandial TG elevation caused by solid food was lower than that of liquid food (101). A range of metabolic, physiological, or functional disorders may occur transiently after ingestion of a high-energy diet rich in fat, and the process is strongly associated with postprandial TG (102). Postprandial CM-TG response increased with an increase in dietary fat content in normal weight and obese individuals (79). The remnant lipoprotein (RLP) particles containing apoB100 and apoB48 promote the formation and development of AS, and the intervention of postprandial RLP interferes with this (103).

A systematic review and meta-analysis of the effects of fat content on postprandial TG response after an oral fat tolerance test showed that there was no difference in response to saturated fatty acids (SFA) and unsaturated fatty acids within 4 h; a lower response to polyunsaturated fatty acids occurred within 8 h, and only a trend was observed for monounsaturated fatty acids, indicating that the postprandial TG response stimulated by different fatty acids is not enough to distinguish in fat tolerance tests shorter than 8 h (104). Studies in patients with type 2 diabetes showed that TG, TG-AUC, and TRL-TG levels improved after a fat tolerance test in patients receiving a Mediterranean diet rich in olive oil for 3 years (80). A research on the chain length of SFA showed that a diet rich in medium-chain SFA like coconut oil had the advantage in reducing postprandial TG and had the potential to improve postprandial lipemia (81). A study of patients with metabolic syndrome found that a diet rich in polyphenols reduced postprandial VLDL content and increased intermediate-density lipoprotein-cholesterol content, and the composition of modified LDL particles changed (82). A high monounsaturated fatty acid diet may have cardioprotective effects in patients with metabolic syndrome by improving postprandial oxidative stress (83).

Postprandial lipid response varies with different dietary types. Hansson et al. studied the effects of different dairy products with similar fat content on postprandial TG of healthy adults. It was found that TG increment AUC of sour cream was 61% (*p* < 0.01), 53% (*p* < 0.01), and 23% (*p* = 0.05) higher than that of whipped cream, butter, and cheese, respectively, indicating that the change of dietary structure may reduce postprandial lipemia (84). In addition, tart cherry has the ability to increase antioxidant activity and reduce TG level after HFM (85) (Table 1).

**Table 1:** Studies of non-medicinal intervention of postprandial lipemia against AS.

**Intervention**	**Method**	**Subjects**	**Targets**	**Effect**	**Reference**
Diet	Breakfasts containing 10 vs. 40 g fat	Normal-weight and obese men	CMRF, plasma LPS transporters, IL-6, and NF-*k*B translocation↑	LPS handling in plasma through CM and LBP seems critical in driving the acute inflammatory response	Vors et al. (79)
	Mediterranean diet rich in olive oil	T2D patients	TG, TG-AUC, TRLs-TG↓	Postprandial TG, TG-AUC, TRLs-TG compared with baseline showed an improvement	Gomez-Marin et al. (80)
	Short, medium and long-chain SFA	Healthy men	TC, HDL, LDL, TG	Medium chain SFA resulted in lower postprandial TG	Panth et al. (81)
	Diet naturally rich in polyphenols	Individuals with high cardiometabolic risk	CM, VLDL1, VLDL2, IDL, LDL, HDL	The composition of LDL particles was modified	Della et al. (82)
	HMUFA	Subjects with the MetS	Postprandial GSH, GSH/GSSH ratio ↑; lipid peroxide, protein carbonyl, SOD activity and plasma H_2_O_2_↓	HMUFA diet improves postprandial oxidative stress in patients with the MetS	Perez-Martinez et al. (83)
	Sour cream, whipped cream, butter, and cheese	Healthy adults	TG increment AUC	TG increment AUC of sour cream was higher	Hansson et al. (84)
	Tart cherry	Normal weight men	ORACFL↑; TG↓	Tart cherry consumption alone or in combination with prior exercise enhanced postprandial antioxidant capacity	Polley et al. (85)
Exercise	CIRC or COMB	Physically active men	TG-iAUC↓	Both 2 exercise designs can reduce PPL	Farinha et al. (86)
	HIIE	Overweight/obese men	TG-AUC↓	Acute submaximal HIIE effectively reduced PPL	O'Doherty et al. (87)
	MI-exercise and HI-exercise	Young men	TG↓, TG-iAUC↓in HE, TBARS ↓, FMD ↑than rest	HI-Exercise was more effective in reducing TG-iAUC, MI-Exercise attenuated the susceptibility of postprandial oxidative damage	Lopes et al. (88)
	Walked for 1 h	Healthy young men	TG↓	Brisk walking effectively reduced postprandial TG	Chiu et al. (89)
	Walked at 60% VO_2peak_ to expend≈5 kcal/kgbw 1 h	Young adults	There were no differences between EX and CON groups	Moderate intensity exercise did not mitigate PPL nor inflammatory response	Teeman et al. (90)
	HIIE	Adolescents	FMD, PRH↑	HIIE protected the postprandial vascular function	Bond et al. (91)
	Two, 2-day main trials (control and exercise)	Adolescent boys	FMD↑, TG concentration vs. time curve↓than control	Repeated 6s maximal cycle sprints improved postprandial endothelial function and TG	Sedgwick et al. (92)
	HIIR	Healthy, active boys	PPL, TG↓	Low-volume HIIR attenuates postprandial TG in adolesents	Thackray et al. (93)
	Intense intermittent exercise, or moderate continuous exercise	Healthy men	TG↓ in both groups, VLDL↓ only in intense intermittent exercise group	Energy expenditure of 500 kcal exercise before HFM reduced PPL	Ferreira et al. (94)
	HIIE and 30 min of brisk walking	Men	TG-AUC, TBARS, protein carbonyls ↓	HIIE attenuates postprandial TG and oxidative stress markers	Gabriel et al. (95)
	HIIT	Physically active young men	FFA uptake ↑	HIIT enhanced clearance of FFA	Wilhelmsen et al. (96)
	HIT or MCT	Healthy, inactive adults	TG-AUC in HIT↓ than MCT, FMD in HIT↑ than MCT	Supervised exercise training mitigated endothelial dysfunction, HIT may be better than MCT	Ramirez-Velez et al. (97)
	Regular aerobic or resistance exercise	Healthy participants	FMD↓in sedentary adults, regular aerobic or resistance exercisers not, and hs-CRP↓, SOD↑	Exercisers may have favorable postprandial metabolic responses or lower inflammation and oxidative stress	Das et al. (98)

*AS, atherosclerosis; TG, triglyceride; CMRF, chylomicron-rich fraction; LPS, lipopolysaccharides; IL-6, interleukin- 6; NF-kB, nuclear factor kB; CM, chylomicrons; T2D, type 2 diabetes; AUC, area under the curve; TRLs, TG-rich lipoproteins; SFA, saturated fatty acids; AUC, under the curve HMUFA, High-mono-unsaturated-fatty-acid; MetS, metabolic syndrome; GSH, reduced glutathione; GSH/GSSH, oxidized glutathione; SOD, superoxide dismutase; ORACFL, oxygen radical absorbance capacity; CIRC, circuit combined exercise; COMB, traditional combined exercise; HIIE, high intensity interval cycling exercise; HIIR, high-intensity interval running; PRH, peak reactive hyperaemia; HIIT, high intensity interval training; FFA, free fatty acid; hs-CRP, hypersensitive-c-reactive-protein*.

#### Exercise

Numerous studies have shown that exercise can have a beneficial effect on postprandial lipemia (86–88, 105). Postprandial lipemia can be reduced by a single exercise (89). Exercise intensity and duration and other factors affect the improvement of postprandial lipemia (90). Exercise intensity is important for the protection of vascular function in adolescent boys between 12 and 15 years of age (91). Studies have demonstrated that adolescent boys between 12.5 and 14.1 years of age who repeatedly sprint for a short time have lower postprandial TG and FMD levels than those who do not exercise (92). Acute high-intensity interval running also reduced postprandial TG in adolescent boys age 11.3–12.9 years (93). Adults who regularly exercise had lower postprandial TG and lower oxidative stress levels such as TBARS than inactive adults, which reduces the negative postprandial changes affecting vascular health (51). Long-term exercise may alleviate postprandial lipid or oxidative stress in older adults with chronic diseases or those with elevated fasting label values (106). In addition, moderate- to high-intensity brief exercise can reduce postprandial TG, while high-intensity interval exercise reduces postprandial free fatty acid, thiobarbituric acid reactive substances, and protein carbonyl oxidative stress markers (94–96). Furthermore, high-intensity training may be more effective than moderate continuous training in reducing vascular injury (97).

There are many mechanisms involved in exercise intervention of postprandial lipemia. Regular aerobic exercise protects endothelial function after a high-fat mixed meal (98). A study to evaluate the effect of 4 h interrupted sitting with 5 min stair climbing after an HFM on vascular found that FMD decreased from 9.41 ± 2.61 to 10.34 ± 3.30% after exercise, indicating that the improvement of lifestyle can prevent postprandial vascular dysfunction. Exercise increases fat oxidation after meals (107, 108). McAllister et al. (109) explored the effect of acute moderate resistance exercise on alleviating postprandial oxidative stress and found that the AUC of advanced oxidation protein products at 4 h after meal was significantly lower than that at rest, and total nitrate/nitrite increased, illustrating that acute resistance exercise can reduce postprandial oxidative stress (Table 1).

### Medicinal Intervention

#### Statin

Drugs related to lipid metabolism also affect postprandial blood lipids (110). Statins are considered an effective method to prevent and treat CVD because they can significantly reduce the level of TG (11). In obese and diabetic patients, postprandial FMD was improved by statins (111–113). Changes in postprandial lipoprotein metabolism after statins have been studied (114), and atorvastatin significantly reduced postprandial TG, TRL apoB48, VLDL, and IDL apoB100 levels in T2D patients with high TG levels (115). However, it is still controversial whether statins can reduce the level of apoB48 in patients with hypertriglyceridemia (110).

#### Fibrate

Fibrates are agonists of peroxisome proliferator-activated receptor α (PPAR-α), regulating lipoprotein metabolism *via* transcription factors. Fibrates have advantages in reducing fasting and postprandial TG and TRL remnant particles (7). Yamashita et al. reported that treatment with pemafibrate, a novel selective peroxisome proliferator-activated receptor α modulator (SPPARMα), enhances reverse cholesterol transport and improves postprandial hyperlipidemia in patients with dyslipidemia (116). Pemafibrate was demonstrated to improve liver function test values and is unlikely to enhance serum creatinine or reduce estimated glomerular filtration rate (117). Sairyo et al. (118) reported that pemafibrate attenuates postprandial hypertriglyceridemia by suppressing the postprandial increase of CM and the accumulation of CM remnants more effectively than fenofibrate in mice. Compared with conventional fibrates, pemafibrate has a better benefit–risk balance, which can be applied to patients who are difficult to use existing fibrates such as patients taking statins or with renal insufficiency (119).

#### Ezetimibe

Ezetimibe is a novel drug for the treatment of dyslipidemia, which selectively inhibits cholesterol absorption by inhibiting Niemann–Pick C1-like protein (NPC1L1). Ezetimibe reduced the postprandial TG-AUC and apoB100 concentration and reduce postprandial endothelial dysfunction (120). In type IIB hyperlipidemia patients, ezetimibe significantly reduced fasting TG, LDL-C, apoB48, and apoB100 levels as well as postprandial TG and apoB48 (121). It is reported that ezetimibe can improve TG and endothelial function in patients with CHD and hypertriglyceridemia during statin treatment (122). Ezetimibe combined with statins reduced the secretion of apoB48, which may be achieved by affecting the metabolism of TRL in intestinal cells (123). In addition, ezetimibe combined with statins reduced the incidence of long-term cardiovascular end events in patients with acute coronary syndrome (124). Therefore, the study of statins combined with ezetimibe to improve postprandial blood lipid levels is worthy of attention.

#### Omega-3 Fatty Acids

Tinker et al. (125) proposed that omega-3 fatty acids may reduce the postprandial TRL apoB by inhibiting the synthesis and secretion of apoB in the liver and intestine. In middle-aged and older adults >40 years, it was found that a high-fish diet can inhibit the production of apoB100 and promote its catabolism to reduce the level of TRL apoB48 after meals (126). Omega-3 fatty acid supplementation significantly inhibited postprandial TG and improved postprandial endothelial dysfunction in healthy subjects (127). In addition, it enhanced the clearance of CM-TG by increasing LPL activity (128). Postprandial TG, apoB48 total AUC, and VLDL apoB100 total AUC were reduced by omega-3 fatty acids in patients with familial hypercholesterolemia (129).

#### Niacin

The effect of niacin on decreasing postprandial TG may be achieved by limiting FFA and inhibiting lipoprotein synthesis (130, 131). In healthy subjects, taking 2 g of extended-release niacin 1 h before an HFM effectively reduced the incremental AUC of TG and FFA and inhibited postprandial triglyceridemia, which may be the result of a significant restriction of FFA (132). However, the negative effects of niacin were reported in HPS2THRIVE and AIM-HIGH trials, which means its clinical application needs further study (133, 134).

#### Others

Recently, proprotein convertase subtilisin/kexin type 9 (PCSK9) has been regarded as an endogenous inhibitor of LDL-C, and anti-PCSK9 monoclonal antibodies have been used for treating hypercholesterolemia (135, 136). Chan et al. reported the effect of subcutaneous injection of PCSK9 inhibitor with evolocumab for 8 weeks on postprandial TRL in healthy adult men. Evolocumab reduced the total area under the curve VLDL-apoB100 (*p* < 0.001), but did not significantly change the kinetics of apoB48 (137).

ApoCIII regulates TG metabolism by inhibiting TG hydrolysis. It has been reported to predict postprandial hypertriglyceridemia independently (138). Evidence showed that apoCIII not only inhibited LPL activity but also restrained the removal of TRLs by LPL-independent pathways (139). ApoCIII inhibitors may be a novel option for the treatment of severe hypertriglyceridemia by effectively reducing plasma TG.

Traditional Chinese medicine is also widely used in the intervention of atherosclerosis. Zhu et al. found that Ilexgenin A (IA) extracted from *Ilex hainanensis* can induce endothelial cells to produce NO, reduce the production of inflammatory cytokines and reactive oxygen species, and improve endothelial dysfunction (140, 141). In a randomized controlled crossover trial with healthy subjects, the experimental group received HFM and a *platycodi radix* beverage (AP). The control group received HFM and a placebo. Blood samples were collected at 0, 2, 4, and 6 h after a meal, and TG and lipoprotein lipase quality were analyzed. The results showed that the plasma lipoprotein lipase quality in the AP group increased significantly at 6 h, and VLDL-TG decreased significantly. It is suggested that a *platycodi radix* beverage may improve postprandial TG response, reduce postprandial lipemia, and decrease the risk of AS by improving the quality of lipoprotein lipase (142).

## Conclusion And Perspective

Fasting lipemia is a diagnostic standard for hyperlipidemia; the body is in the postprandial state most of the time, so the effect of postprandial lipemia on the human body is worthy of attention. The increase in postprandial TG is the main manifestation of postprandial hyperlipidemia. The relationship between diurnal TG pattern and postprandial blood lipid is worthy of further exploration. The results show that TG has a larger diurnal organism than TC, HDL-C, LDL-C, and other lipoproteins. Therefore, postprandial lipemia detection may reflect the true level of TG. A meta-analysis of 113 studies showed that the level of TG 4 h after the fat tolerance test was the most representative (143). Research showed that compared with the detection of postprandial TG, increment and total area under the curve before and after the meal tested hourly for 6 h and subjects that had blood drawn before and 4 h after meals without restriction of activity had results similar to those of the previous test, which provided a simple method for the detection and application of postprandial lipemia (144). Maraki et al. (145) have also shown that postprandial lipemia may be accurately described by the OFTT with reduced frequency of blood sampling.

The mechanism of AS induced by TRL needs to be further studied on the basis of the postprandial TG curve, and the standardized fat load test still needs improvement (143, 146). CVD caused by postprandial dyslipidemia has been reflected in many clinical cases. The mechanism of postprandial lipemia affecting the occurrence and development of AS may be endothelial dysfunction, oxidative stress, inflammation, or others (147–151). The accumulation of hepatic apoB100 and circulating intestinal apoB48 TRL are characteristic phenomena of postprandial lipemia, and both of them are involved in the development of atherosclerotic plaque (100). It has been suggested that postprandial RLP and remnant-like particles are sensitive and important in the risk estimates of AS (152). Many uncontrollable factors [such as, genetic background (153), age, sex, and menopausal state], as well as lifestyle (diet, physical activity, smoking, and drug use), can affect postprandial lipids (9, 87, 154, 155). Non-medicinal therapy including diet adjustment and exercise has beneficial effects on CVD (156). The mechanism of anti-AS of dietary intervention on postprandial lipemia is mainly inflammatory change, while exercise shows more improvement in postprandial oxidative stress. The anti-atherosclerotic mechanism of medicinal interventions such as, statins, fibrate, ezetimibe, and omega-3 fatty acids on postprandial lipemia is mostly manifested as improvement of vascular endothelial function. As novel intervention methods, pemafibrate, PCSK9 inhibitors, and apoCIII inhibitors are the hot spots of current research.

The joint detection of fasting lipids and postprandial lipids is helpful for clinicians to collect the lipid metabolism of patients more comprehensively, evaluate the risk of cardiovascular disease more accurately, and play a complementary role in diagnosis and treatment. Therefore, postprandial lipemia detection is recommended for clinical application as a Supplementary Material. Recently, postprandial lipemia was found to be well-suited to be measured by quantile-dependent expression, which significantly increases the exposure of individual's gene phenotypes in plasma TG (140). Further research on postprandial dyslipidemia and postprandial hyperlipidemia and an in-depth exploration of its mechanism and intervention measures may provide a new direction for the regulation of public health. Gut microbiota is closely related to lipid metabolism, and some studies have indicated that intestinal microorganisms may affect lipid metabolism as environmental factors (157). There are few reports on the relationship between postprandial lipemia and gut microbiota, but animal studies have shown that gut microbiota increases the level of epoxyeicosatrienoic acid and improves CVD by inhibiting postprandial soluble epoxide hydrolase (158). Whether intervention of gut microbiota can reduce postprandial lipemia, and therefore against AS, may be worthy of further study.

## Author Contributions

YZ, LL, and MW designed the article and wrote the manuscript. SY, GL, and LP created the table. CG and YW designed the figure. DL and RZ commented on the manuscript. All authors approved the manuscript for publication.

## Conflict of Interest

The authors declare that the research was conducted in the absence of any commercial or financial relationships that could be construed as a potential conflict of interest.
